# Association between social determinants of health and systemic lupus erythematosus: a nationally representative analysis of 2017–2021 data

**DOI:** 10.1038/s41598-025-13071-7

**Published:** 2025-08-08

**Authors:** Ami Vyas, Steven Cohen, Christine Eisenhower

**Affiliations:** 1 College of Pharmacy, Department of Pharmacy Practice and Clinical Research, University of Rhode Island, 7 Greenhouse Road, Kingston, 02881 RI USA; 2 College of Health Sciences, Department of Health Studies, University of Rhode Island, Kingston, RI USA

**Keywords:** Systemic lupus erythematosus, Medical expenditure panel survey, Social determinants of health, Logistic regression, Socioeconomics, Rheumatology, Risk factors

## Abstract

Limited US recent data is available on the prevalence of systemic lupus erythematosus (SLE) by patient’s social determinants of health (SDOH). Careful assessment of individual SDOH that affects SLE is crucial, as such evidence could help improve care and hence reduce health disparities for patients with SLE, especially for those who are most vulnerable and at the highest risk of poor outcomes. We estimated the prevalence of systemic lupus erythematosus (SLE) overall and by patient’s social determinants of health (SDOH), and also explored the associations between SDOH and SLE. We conducted a population-based cross-sectional study using Medical Expenditure Panel Survey 2017–2021 data. Patients with SLE were those with both SLE diagnosis and either had SLE-related medication use and/or visited a rheumatologist in the survey year. SDOH domains included economic stability, education, healthcare access and quality, social and community context, and neighborhood and built environment. Average annual prevalence of SLE by SDOH was determined. Separate logistic regressions were used to examine the association between each SDOH and SLE, controlling for confounders. Average annual SLE prevalence was 199 per 100,000 US adults (95% confidence intervals:170–224). In the economic stability domain, those with low family income showed higher odds of SLE than those with high family income (adjusted odds ratio (AOR) = 2.779, *p* < 0.05). Within the social and community context and neighborhood and built environment domains, non-Hispanic Black patients (AOR = 2.429, *p* < 0.05) and patients with any psychological distress (AOR = 2.127, *p* < 0.05) had higher odds of SLE, than their respective counterparts, respectively. Within the healthcare access and quality domain, those with Medicaid insurance had higher odds of SLE (AOR = 2.540, *p* < 0.05) than those with private insurance. Also, patients in the highest SDOH burden quartile had higher odds of SLE (AOR = 2.039, *p* < 0.05) than those in the lowest SDOH burden quartile. We identified several subgroups of patients especially those with higher social disadvantage and a higher SDOH burden. The prevalence of SLE increased with a higher SDOH burden.

## Introduction

Systemic Lupus Erythematosus (SLE) is a multifactorial chronic autoimmune disease, with a multitude of clinical manifestations and involvement of one or more body organs^1^. The prevalence of SLE ranges from 73 to 178 per 100,000 in the United States (US)^2^. These prevalence estimates are obsolete and were derived from a small group of homogenous patient populations from specific geographic locations not representative of the US population. A meta-analysis pooled data from the Centers for Disease Control and Prevention National Lupus Registry network including five geographic locations and reported the SLE prevalence of 73 per 100,000 during 2002–2009^3^.

Etiology, prognosis, and treatment of chronic diseases are influenced by several factors^4^. Certain individuals are more prone to severe diseases and may also struggle to access preventive care or basic primary treatment due to impediments not directly associated with the healthcare setting, known as social determinants of health (SDOH)^5^. The World Health Organization identifies SDOH as the major cause of health disparities and increasing healthcare costs^5^. Indeed, SDOH can affect approximately 50% of health outcomes^6^, with socioeconomic factors such as poverty, employment, and education having the most significant impact^6^.

Disparities in SLE are not well explained, and empirical evidence suggests that generic and biological causes do not fully explain variability in the disease risk^7^. Recently, there has been an emphasis on investigating the links between SDOH and SLE^8–10^. Established risk factors of SLE such as race and ethnicity are documented^11,12^. Additionally, transportation challenges^10^, medication and mental healthcare access^10^, and economic insecurity were found to be highly prevalent in patients with SLE^10,13^, but the data were limited to specific US geographic areas.

Careful assessment of individuals’ social, economic, and environmental conditions that affect SLE is crucial, as such evidence may improve care and reduce health disparities for patients with SLE, especially those most vulnerable and at the highest risk. Hence, the purpose of this study was to determine the overall prevalence of SLE in the US using the most recent years of data, and also examine the prevalence of SLE by factors belonging to the SDOH domains. Most importantly, the associations between SDOH and SLE were explored to identify the significant SDOH factors that are associated with SLE prevalence.

## Methods

### Study design & data source

A population-based cross-sectional study was conducted using publicly available Medical Expenditure Panel Survey (MEPS) survey data from 2017 to 2021. The MEPS is a nationally representative, population-based household survey of the US non-institutionalized civilian population and is co-sponsored by the Agency for Healthcare Research and Quality (AHRQ) and the National Center for Health Statistics (NCHS). MEPS collects patient-level and household-level data on respondent’s demographic and socioeconomic characteristics, access to care, health status, clinical diagnosis and related payments, and healthcare utilization and expenditures from five rounds of interviews conducted over a two-year period^14^. The medical conditions files in MEPS contain information on medical conditions and coded to International Classification of Disease, 10th Edition, Clinical Modification (ICD-10-CM) codes^15^. We used household component files, medical conditions files, prescribed medications files, food security files, and office-based medical provider visit, and outpatient visit files for our study.

### Study population

The study population comprised of non-institutionalized US adults who did not die during the survey calendar year. Similar to a previously published MEPS study^11^, we identified adults with SLE as those with SLE and either who had SLE-related medication use and/or visit to a rheumatologist in the survey calendar year. The ICD-10-CM code of M32 from the medical conditions files was used to identify individuals who reported having been diagnosed with SLE. The SLE-related medications included antimalarials, oral or intravenous/ injectable corticosteroids, biologics, calcineurin inhibitors, and immunosuppressants, identified in consultation with the clinical pharmacy faculty, and after identifying the medications from the 2019 European Alliance of Associations for Rheumatology (EULAR) and 2021 American College of Rheumatology recommendations for the treatment of SLE^16–18^. A visit to a rheumatologist was identified from the office-based and outpatient visit files.

### Study measures

#### Prevalence of SLE

Prevalence of SLE was estimated as the weighted proportion of the US population with SLE and reported per 100,000 US adults.

#### Social determinants of health

SDOH are the social and economic conditions in which people are born, grow, work, and age^5^. Together, these conditions can affect the overall health and quality of life of individuals and communities and can also begin the onset of chronic health issues, thereby influencing disease etiology and prognosis^19^. SDOH are categorized into five domains: economic stability, education, health care access and quality, social and community context, and neighborhood and built environment^20^. In our study, economic stability was assessed by examining the patient’s employment status, poverty level, family income level, use of food stamps (yes, no), and food insecurity. Similar to that used in a previously published study^21^, food insecurity variable was created by summing the responses to ten survey questions on food insecurity with the value of 1 for each survey question response indicating ‘food insecurity’ and 0 indicating ‘no food insecurity’. After getting the sum of responses to 10 questions, food insecurity was categorized as full food security (sum score of 0), marginal food security (sum score of 1–2), and low (sum score of 3–5) to very low (sum score of 6–10) food security^21^. Education was assessed by examining the patient’s education level. Health care access and quality was assessed by examining the type of patient’s health insurance coverage (private, Medicare, Medicaid, uninsured), access to primary care, access to health care, transportation issues to visit primary care, and satisfaction with the usual source of care provider. Similar to a previously published study^22^, the patient’s access to primary care was determined based on the response to the survey question of whether the patient had a usual source of care (yes, no) in the survey calendar year. The patient’s access to health care was determined based on whether the patient had unmet medical needs and unmet prescription medication needs, similar to that used previously^23^. Transportation issues to visit primary care was assessed by examining the amount of time the patient needed to travel to their usual source of care provider. Patient’s satisfaction with their usual source of care provider was determined based on the patient’s responses to four survey questions: “Does the usual source of care provider ask [i] about prescription medications and treatments other doctors may give them; [ii] the patient to help make decisions between a choice of treatments; [iii] present and explain all options to the person; and [iv] speak the person’s language or provide translator services?”^24^.

Social and community context was assessed by examining patient’s social support status, language spoken (English, non-English), geographic location in the US, time lived in the US or nativity, and race/ethnicity (i.e., non-Hispanic White, non-Hispanic Black, Hispanic, others). The ‘other’ race/ethnicity group included all individuals from other and/or multiple races/ethnicities. We used a patient’s marital status as a proxy for examining social support status. Neighborhood and built environment were assessed by examining patient’s health behaviors including physical activity and smoking status, and non-specific psychological distress. Smoking is associated with country social and physical environment factors^25^, and living in deprived neighborhoods was associated with lower exercise time^26^. Also, individuals who live in deprived neighborhoods experience more stress than those living in non-deprived neighborhoods^27^. Non-specific psychological distress was assessed using the responses to the six survey questions from the validated Kessler Index (K6) scale^28^. The response scores were summed for a total possible score range of 0 to 24 based on which psychological distress was categorized as 0 (no psychological distress), 1–12 (without serious psychological distress), and 13–24 (serious psychological distress)^29,30^. The appendix 1 provides a detailed description of SDOH and which survey questions were used to identify these from MEPS and their categorization.

Similar to a published study^31^, we generated an aggregated SDOH score to examine the association between cumulative SDOH burden and SLE. For doing so, we assigned a value of ‘0’ if the SDOH was favorable and ‘1’ if the SDOH was unfavorable. For instance, use of food stamps is unfavorable and hence was assigned a value of ‘0’ for the ‘use of food stamps’ variable if patients who used food stamps in the survey year. Similarly, the response of ‘no’ for patient’s access to primary care is unfavorable and hence was assigned a value of ‘0’. We summed the individual values for each SDOH to generate an aggregate SDOH score. Based on the distribution of the SDOH scores, we categorized SDOH score into four categories (low for 0–3 score, moderate for 4–5 score, high for 6–7 score, and very high for ≥ 8 score), with the highest score category representing the highest cumulative SDOH burden.

#### Covariates

We assessed age, sex, physical and mental health comorbidities derived from the medical conditions files using the respective ICD-10 CM codes, and visit to general practitioner in the survey calendar year. The co-occurring physical conditions included deformities and joint disorders, arthritis, hypertension, asthma, heart disease, stroke, chronic obstructive pulmonary disease, gastroesophageal reflux disease, sleep disorders, migraine, osteoporosis and related diseases, and diabetes, while the mental health/neurologic comorbidities included depression, anxiety, mood disorders, and schizophrenia.

### Statistical analyses

Descriptive statistics were used to describe the study population (means and standard deviations and/or frequencies, where relevant). The overall prevalence of SLE in the US was calculated annually from 2017 to 2021, and also for 2017–2021 pooled data. Chi-square tests were used to compare differences among SLE and non-SLE patients by key patient characteristics and SDOH. The prevalence of SLE across each SDOH variable and cumulative SDOH burden was estimated.

To examine associations between SLE and each SDOH, we adopted a cross-sectional, matched comparative cohort study design due to the lower prevalence of SLE. The cases comprised patients with SLE, while the comparative cohort comprised individuals without SLE. Each patient with SLE was matched to eight controls by age, sex, and MEPS survey calendar year. To determine the balance between cases and controls after matching, we estimated the standardized mean differences (SMD) of the variables on which matching was performed and used the threshold of SMD < 0.1 to assess a good balance between the cases and controls groups^32,33^. Adjusted logistic regressions were conducted to examine the association between each SDOH and SLE, and also between the cumulative SDOH burden and SLE, after adjusting for confounders.

We adjusted for the complex survey design of MEPS in order to generate nationally representative estimates and a p-value of < 0.05 was considered statistically significant. All analyses were conducted using survey procedures in SAS version 9.4 (Cary, NC, US).

## Results

**Fig. 1:**
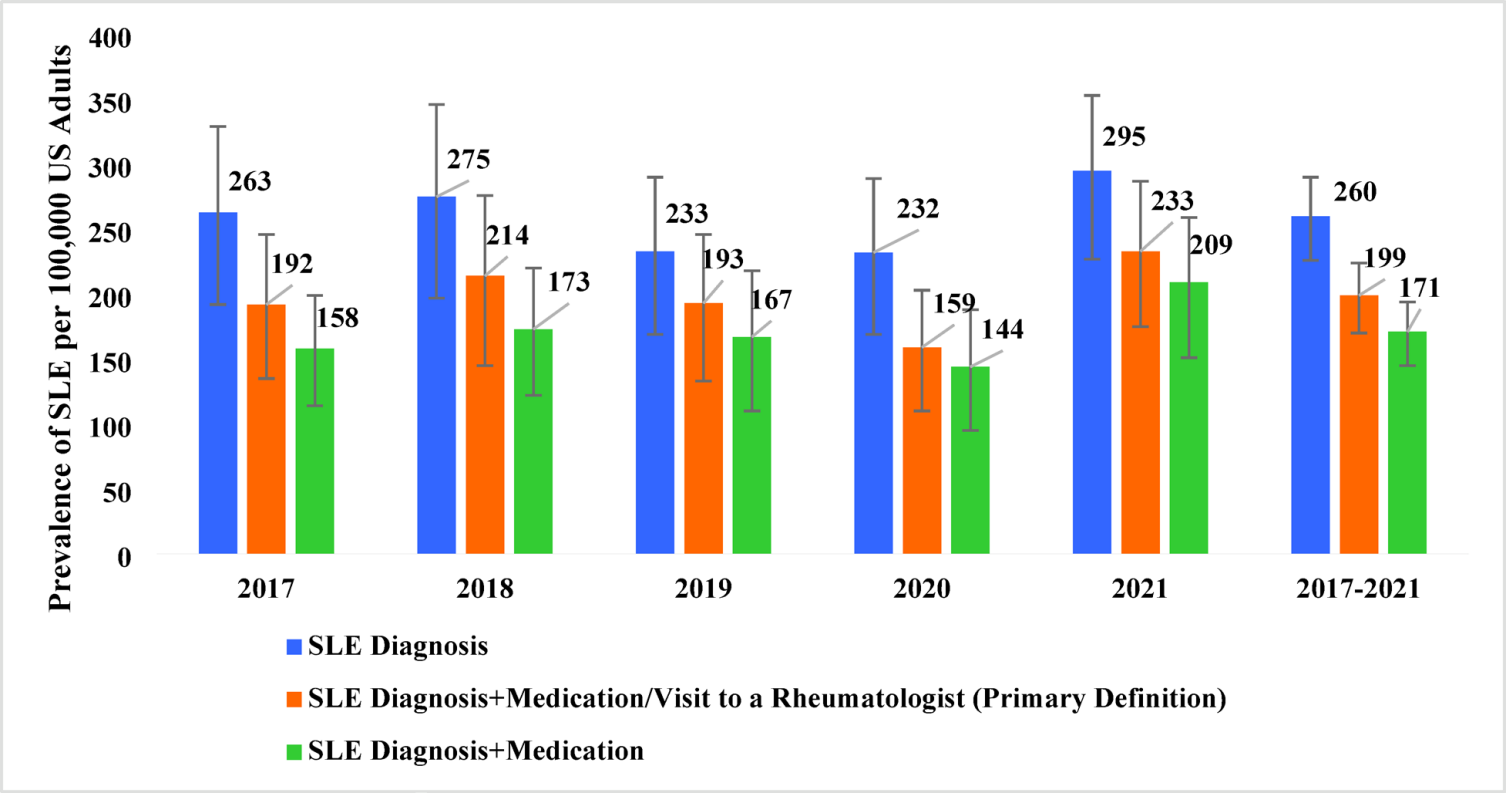
Prevalence of systemic lupus erythematosus in the United States. SLE: systemic lupus erythematosus.

The final weighted analytic sample for 2017–2021 data comprised approximately 251 million annualized non-institutionalized US adults, out of which 0.20% had SLE during the survey year. The pooled weighted average annual prevalence of SLE using the primary definition of SLE was 199 per 100,000 adults (95% confidence intervals (CI): 170, 224) (Fig. 1). The prevalence of SLE increased from 192 per 100,000 adults in 2017 to 233 per 100,000 adults in 2021, with a slight decline reported in 2020. The same trend continued for the SLE prevalence estimates using other definitions of SLE.

For the pooled 2017–2021 data, the weighted average annual prevalence of SLE was two times higher in adults aged 55–64 years compared to those aged 18–39 years, almost 13 times higher in women versus men (359 vs. 28 per 100,000 adults), nine times higher in those with 2 or more physical comorbidities compared to those with no comorbidities (703 vs. 76 per 100,000 adults), and almost three times higher in those with mental health comorbidities than those without such comorbidities (Table 1). For the economic stability SDOH domain, SLE prevalence was twice as high among those who used food stamps (355 vs. 182 per 100,000; *p* = 0.0037) and were unemployed, and more than three times in those with very low food security (667 vs. 180 per 100,000; *p* = 0.0002), compared to their respective counterparts. Also, SLE prevalence was significantly higher in those with poor to negative or near poor income compared to those with high income. For the social and community context SDOH domain, SLE prevalence was twice as high among non-Hispanic Black adults than non-Hispanic White adults (367 vs. 177 per 100,000 adults). Within the neighborhood and built environment SDOH domain, adults with no physical activity had twice the prevalence of SLE than those who had reported physical activity (257 vs. 140 per 100,000 adults). Also, adults with psychological distress had higher prevalence of SLE than those without psychological distress (*p* < 0.0001). Within the healthcare access and quality SDOH domain, those with Medicaid insurance had a higher prevalence of SLE than those with private insurance (398 vs. 161 per 100,000; *p* < 0.0001), while those with usual source of care had higher SLE prevalence than those without a usual source of care (250 vs. 73 per 100,000 adults; *p* < 0.0001). Moreover, those with unmet prescription needs had higher SLE prevalence compared to those with no unmet prescription needs (539 vs. 189 per 100,000 adults). With regard to the cumulative SDOH burden, those with very high SDOH burden had more than three times the prevalence of SLE than those with low SDOH burden (360 vs. 113 per 100,000 adults; *p* < 0.0001) (Table 1).

**Table 1:** Description of demographic characteristics and social determinants of health of the study population and prevalence of systemic lupus erythematosus by each patient characteristic.

Variables		SLE (%)	No SLE (%)	Prevalence of SLE Per 100,000 US adults	95% confidence intervals
Age (mean (SE))**		53.1 (1.48)	48.0 (0.16)		
Age**	18–39	25.3	37.5	134	82–187
	40–54	21.7	24.2	178	111–246
	55–64	26.4	16.7	314	198–430
	65,+	26.6	21.6	244	161–328
Sex***	Females	93.2	51.6	359	290–428
	Males	6.8	48.4	28	7–49
Visit to a general practitioner	Yes	30.3	28.0	215	149–282
	No	69.7	72.0	192	148–237
Physical comorbidities^α^ ***	0	21.1	55.3	76	46–105
	1	37.8	33.1	227	157–297
	2,+	41.1	11.6	703	511–895
Mental health comorbidities^£^ ***	0	64.2	83.7	153	113–192
	1,+	35.8	16.3	436	296–575
Social determinants of health					
Economic stability domain					
Food security **	Full food security	81.4	88.5	180	135–225
	Marginal food security	4.7	5.0	181	49–313
	Low food security	4.3	3.7	227	12–441
	Very low food security	9.6	2.8	667	270-1,064
Use of food stamps**	No	82.9	90.4	182	142–222
	Yes	17.1	9.6	355	209–501
Employment status**	Employed	52.2	69.5	149	108–191
	Not employed	47.8	30.5	311	228–395
Poverty level **	Poor/negative income	18.1	10.4	347	200–494
	Near poor income	8.9	3.7	478	166–790
	Low income	9.9	11.9	165	95–235
	Middle income	24.4	28.4	170	117–224
	High income	38.7	45.6	169	117–221
Family income level**	≤$25,000	30.2	17.5	343	229–457
	$25,001-$50,000	20.3	19.7	204	130–279
	$50,001-$100,000	26.5	29.7	177	108–246
	>$100,000	23	33.1	138	84–193
Education domain					
Education level	≤ High school	41.4	40.6	202	142–263
	1–2 years of college	21.2	21.6	196	117–274
	3–4 years of college	20.3	24.4	165	98–233
	5,+ years of college	17.1	13.4	254	128–379
Social & community context domain					
Race/ethnicity*	Non-Hispanic White	55.5	62.4	177	132–221
	Non-Hispanic Black	22	11.9	367	189–544
	Hispanic	12.2	16.6	146	85–207
	Other races or multiple races	10.3	9.1	224	83–365
Marital status	Married	46.3	51.5	179	131–227
	Divorced/Widowed/Separated/Never married	53.7	48.5	220	162–278
Geographic location	Northeast	23.3	17.5	265	144–385
	South	37.9	38.0	198	136–261
	Midwest	23.4	20.8	223	141–305
	West	15.5	23.8	129	76–183
Language spoken	English	82.1	79.9	204	159–249
	Non-English (Spanish or other)	17.9	20.1	177	93–261
Time lived in the US / Nativity	Born in the US	83.2	82.3	201	159–242
	< 1–10 years	1.7	3.2	105	0-256
	> 10 years	15.1	14.5	207	97–317
Neighborhood & built environment domain					
Physical activity ***	No	64.9	50.1	257	200–314
	Yes	35.1	49.9	140	101–179
Smoking status	Smoker	16.5	13.3	245	138–352
	Non-smoker	83.5	86.7	192	150–233
Psychological distress***	No psychological distress	24.2	51.3	94	65–122
	Without serious psychological distress	70.2	45.7	305	239–371
	With serious psychological distress	5.6	3.0	376	125–627
Healthcare access & quality domain					
Health insurance type***	Private < 65 years old	47.1	58.3	161	114–207
	Medicare ≥ 65 years old	25.2	21.2	236	158–313
	Medicaid (all ages)	26.0	13.0	398	254–542
	Uninsured	1.7	7.5	45	0–95
Usual source of care***	Yes	89.4	71.0	250	199–301
	No	10.6	29.0	73	35–111
Transportation time to usual source of care	≤ 30 min	89.9	92.8	193	154–231
	31–90 min	9.0	6.6	269	149–389
	> 90 min	1.1	0.6	384	0-828
Unmet medical needs	Yes	12.9	8.2	313	157–470
	No	87.1	91.8	189	149–228
Unmet prescription needs***	Yes	11.5	4.2	539	305–774
	No	88.5	95.8	184	146–221
Satisfaction with usual source of care provider					
1. Provider asked patient about other treatment	Yes	88.4	87.1	202	162–241
	No	11.6	12.9	179	108–250
2. Provider asked patient to help decide	Yes	75.9	80.4	188	149–226
	No	24.1	19.6	244	164–324
3. Provider explained options to patient	Yes	96.3	97.2	197	158–235
	No	3.7	2.8	265	70–459
4. Provider speaks patient’s language*	Yes	99.1	99.7	197	159–236
	No	0.9	0.3	712	0-1592
Cumulative SDOH burden					
SDOH burden***^§^	Low (0–3)	14.7	25.4	113	63–163
	Moderate (4–5)	20.9	28.7	143	80–205
	High (6–7)	21.7	22.8	186	103–269
	Very high ( > = 8)	42.7	23.1	360	247–473

***p < 0.0001; **0.0001 ≤ *p* < 0.01; *0.01 ≤ *p* < 0.05.

^δ^for primary definition of SLE.

^α^included hypertension, diabetes, deformities and joint disorders, arthritis, sleep disorders, osteoporosis, asthma, heart disease, stroke, gastrointestinal reflux disease, lipedema, chronic obstructive pulmonary disease and related diseases, thyroid disorder, pain, migraine, other soft tissue disorders.

^£^included depression, mood disorders, anxiety, and schizophrenia.

^§^for 2017, 2020, 2021 years only as food security data was available only for these years.

SLE: Systemic lupus erythematosus; US: United States; SE: Standard error; SDOH: Social determinants of health.

**Fig. 2:**
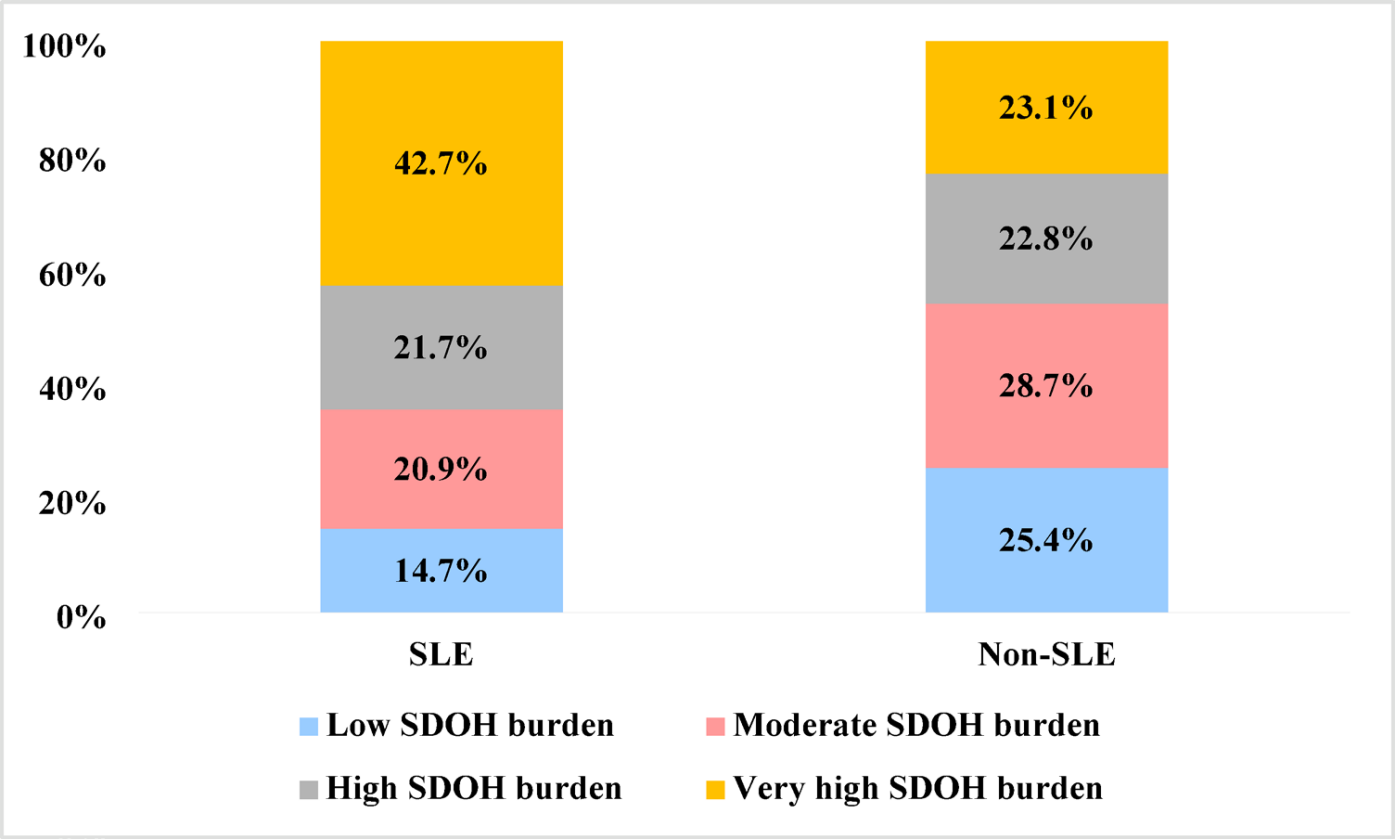
Prevalence of cumulative social determinants of health burden among those with and without systemic lupus erythematosus ^§^*. *p < 0.0001. ^§^for primary definition of systemic lupus erythematosus. SLE: systemic lupus erythematosus; SDOH: social determinants of health.

Figure 2 shows the prevalence of cumulative SDOH burden among those with and without SLE. 65% of patients with SLE had a high to very high SDOH burden compared to about half of those without SLE (65% vs. 46%; *p* < 0.0001). Only 15% of patients with SLE had a low SDOH burden compared to 25% of adults without SLE.

In the adjusted analyses (Table 2), in the economic stability SDOH domain, those with poor family income showed higher odds of SLE than those with high family income (adjusted odds ratio (AOR) = 2.779, 95% confidence interval (CI): 1.029–7.504). Within the social and community context SDOH domain, non-Hispanic Black adults (AOR = 2.429, 95%CI: 1.311-4.500) and Hispanic adults (AOR = 1.616, 95% CI: 0.912–2.864) had higher odds of SLE than non-Hispanic White adults. Also, those living in the Northeast had higher odds of SLE than those living in the West (AOR = 2.612; 95%CI: 1.265–5.393). Within the neighborhood and built environment SDOH domain, patients with any psychological distress (AOR = 2.127; 95%CI: 1.324–3.419) and those who had no physical activity (AOR = 1.677, *p* < 0.05) had higher odds of SLE. Within the healthcare access and quality SDOH domain, those with Medicaid insurance (AOR = 2.540, 95%CI: 1.406–4.589) and with the usual source of care (AOR = 2.427; 95%CI: 1340 − 4.386) had higher odds of SLE than their respective counterparts. With regard to cumulative SDOH burden, adults in the highest SDOH burden category had significantly higher odds of SLE (AOR = 2.039, 95%CI: 1.148–3.622) than those in the lowest SDOH burden quartile (Table 2).

**Table 2:** Association between social determinants of health and systemic lupus erythematosus^§^δ.

		Unadjusted odds ratio	95% confidence intervals	Adjusted odds ratio*	95% confidence intervals
Economic stability domain					
Food security	Full food security	Reference		Reference	
	Marginal food security	1.054	0.475–2.340	0.736	0.316–1.717
	Low food security	1.177	0.403–3.437	0.670	0.204–2.195
	Very low food security	**3.338**	**1.659–6.716**	1.724	0.725–4.101
Use of food stamps	No	Reference		Reference	
	Yes	**2.282**	**1.406–3.702**	1.480	0.841–2.602
Employment status	Employed	Reference		Reference	
	Not employed	**1.778**	**1.118–2.827**	1.124	0.667–1.895
Poverty level	Poor/negative income	**1.920**	**1.100–3.350**	1.235	0.682–2.238
	Near poor income	**4.189**	**1.735–10.114**	**2.779**	**1.029–7.504**
	Low income	1.141	0.590–2.206	0.839	0.411–1.715
	Middle income	1.226	0.695–2.160	1.082	0.602–1.944
	High income	Reference		Reference	
Family income level	≤$25,000	**2.693**	**1.495–4.851**	1.492	0.805–2.764
	$25,001-$50,000	1.772	0.886–3.543	1.264	0.625–2.557
	$50,001-$100,000	1.612	0.854–3.043	1.249	0.657–2.375
	>$100,000	Reference		Reference	
Education domain					
Education level	≤ High school	1.011	0.514–1.988	0.742	0.353–1.558
	1–2 years of college	0.881	0.406–1.913	0.715	0.313–1.636
	3–4 years of college	0.687	0.333–1.415	0.665	0.308–1.436
	5,+ years of college	Reference		Reference	
Social & community context domain					
Race/Ethnicity	Non-Hispanic White	Reference		Reference	
	Non-Hispanic Black	**2.379**	**1.285–4.404**	**2.429**	**1.311-4.500**
	Hispanic	1.307	0.744–2.296	1.616	0.912–2.864
	Other races or multiple races	1.363	0.569–3.266	1.727	0.679–4.394
Marital status	Married	Reference		Reference	
	Divorced/Widowed/Separated/Never married	1.348	0.870–2.091	0.988	0.613–1.592
Geographic location	Northeast	**2.737**	**1.350–5.547**	**2.612**	**1.265–5.393**
	South	**1.908**	**1.026–3.551**	1.619	0.837–3.130
	Midwest	**2.007**	**1.005–4.005**	1.848	0.902–3.786
	West	Reference		Reference	
Language spoken	English	Reference		Reference	
	Non-English (Spanish or other)	1.009	0.577–1.766	1.007	0.344–2.946
Time lived in the US / Nativity	Born in the US	Reference		Reference	
	< 1–10 years	0.674	0.146–3.124	0.903	0.146–5.592
	> 10 years	1.273	0.681–2.378	1.353	0.599–3.059
Neighborhood & built environment domain					
Physical activity	No	**2.035**	**1.426–2.904**	**1.677**	**1.153–2.440**
	Yes	Reference		Reference	
Smoking status	Smoker	1.224	0.710–2.108	1.053	0.582–1.905
	Non-smoker	Reference		Reference	
Psychological distress	No psychological distress	Reference		Reference	
	With any psychological distress (including serious psychological distress)	**2.350**	**1.573–3.510**	**2.127**	**1.324–3.419**
Healthcare access & quality domain					
Health insurance type	Private < 65 years old	Reference		Reference	
	Medicare ≥ 65 years old	**1.655**	**1.012–2.706**	1.096	0.637–1.883
	Medicaid (all ages)	**3.607**	**2.101–6.191**	**2.540**	**1.406–4.589**
	Uninsured	0.491	0.134–1.802	0.502	0.138–1.821
Usual source of care	Yes	**3.188**	**1.734–5.863**	**2.427**	**1.340–4.386**
	No	Reference		**Reference**	
Transportation time to usual source of care	≤ 30 min	Reference		Reference	
	> 30 min	0.748	0.383–1.460	0.893	0.453–1.762
Unmet medical needs	Yes	**1.864**	**1.043–3.331**	1.159	0.620–2.166
	No	Reference		Reference	
Unmet prescription needs	Yes	**2.185**	**1.193-4.000**	1.138	0.595–2.177
	No	Reference		Reference	
Satisfaction with usual source of care provider					
1. Provider asked patient about other treatment	Yes	1.355	0.831–2.207	**1.825**	**1.096–3.039**
	No	Reference			
2. Provider asked patient to help decide	Yes	Reference		Reference	
	No	1.193	0.762–1.869	0.953	0.584–1.556
3. Provider explained options to patient	Yes	Reference		Reference	
	No	1.794	0.727–4.427	1.332	0.439–4.043
4. Provider speaks patient’s language	Yes	Reference		Reference	
	No	2.411	0.323–17.985	2.789	0.306–25.468
Cumulative SDOH burden					
SDOH burden	Low (0–3)	Reference		Reference	
	Moderate (4–5)	1.360	0.767–2.411	1.209	0.686–2.130
	High (6–7)	**1.930**	**1.042–3.577**	1.642	0.861–3.129
	Very high (≥ 8)	**2.900**	**1.711–4.916**	**2.039**	**1.148–3.622**

Statistically significant values with p < 0.05 are in bold.

^δ^for the primary definition of systemic lupus erythematosus.

^§^for 2017, 2020, 2021 years only as food security data was available only for these years.

US: United States; SDOH: Social determinants of health.

*adjusted for physical and mental health conditions, visit to a general practitioner, and the rest of SDOH using the backward selection model.

## Discussion

To the best of the authors’ knowledge, this is the first study that examined the prevalence of SLE by SDOH and also the associations between SDOH and SLE to identify the subgroups of patients who have higher SLE burden, using the nationally representative MEPS data of non-institutionalized US adults. We found that the prevalence of SLE in the US during 2017–2021 was 199 per 100,000 US adults, an estimate substantially higher than reported in prior studies^2,3^, but similar to that published in another MEPS-based study that used 2016–2018 data^11^. Coherent with the published studies, the burden of SLE was found to be significantly higher in females than males^3,11,12^, in the 55–64 years age group^34^, and those with physical and/or mental health comorbidities^35,36^.

The prevalence of SLE was also found to be disproportionately higher in patients with certain unfavorable SDOH. For instance, within the economic stability SDOH domain, US adults with very low food security, who used food stamps, were unemployed, and had poor income had a higher prevalence of SLE than their respective counterparts. Within the social and community context SDOH domain, we found that non-Hispanic Black adults had a higher prevalence of SLE than non-Hispanic White adults, and within the neighborhood and built environment domain, those with no physical activity, with psychological distress and with residence in Northeast had a higher prevalence of SLE. Our findings confirm the prior reports that SLE affects susceptible and less-privileged subgroups^37,38^ and Black individuals^2^. It is well established that lower socioeconomic status and residence in disadvantaged neighborhoods can lead to poor access to food, higher use of food stamps, and unhealthy behaviors. Food insecurity and use of food stamps are strongly associated with increased use of ultra-processed foods^39^, which is one of the risk factors of SLE identified in a large epidemiologic Nurses Health Study^40^. It is also evident that poverty is associated with worse physical activity and functioning^41^. It is likely that SLE patients who live in poverty may not have access to safe and walkable neighborhoods resulting in no physical activity. Contrastingly, it is also likely that due to disease severity which may affect disability status, patients with SLE may have low physical activity. Reports also suggest a strong relationship between psychological distress and SLE^42^, which can result from disease itself and/or from an interplay of other SDOH. Therefore, it is difficult to determine which of the SDOH variables are most likely responsible for the observed associations. However, the findings are generally consistent with the vast body of research on other health conditions with respect to the association between various measures of SDOH and health among patients with SLE.

Within the healthcare access and quality SDOH domain, individuals with Medicaid coverage had a significantly higher prevalence of SLE than those with private insurance coverage, which is consistent with previous research^43^. Medicaid programs serve individuals with low income who are negatively impacted by several social determinants, including lack of affordable housing, employment, food, and transportation to care, which again reflects general social disadvantage on impacting SLE prevalence. Also, those with access to primary care assessed by ascertaining the usual source of care had higher odds of SLE than those without access to primary care. It is likely that patients with SLE because of their condition itself and disease-related manifestations and higher number of comorbid conditions are more likely to have a usual source of care. Reports show that individuals with chronic conditions have a higher likelihood of having a usual source of care^44^. Patient-provider relationships can affect SLE outcomes. Due to their condition, providers can be more likely to ask patients with SLE about other treatments, due to which we found a significant association between that aspect of patient satisfaction with SLE prevalence. Studies have reported that better patient-physician interactions have resulted in improved outcomes in patients with SLE^45^.

Perhaps most notably, our study found a graded increase in the prevalence of SLE with increasing cumulative SDOH burden, indicating that as the number of social disadvantage factors increased, the prevalence of SLE increased as well. Those with ≥ 8 factors of social disadvantage had more than three times the prevalence of SLE than those with ≤ 3 factors of social disadvantage. This association remained significant in the adjusted analyses as well. Hence, our findings emphasize the role of several SDOH and cumulative SDOH as crucial determinants of SLE burden. In fact, we found that 65% of patients with SLE had a high to very high cumulative SDOH burden compared to 46% of patients without SLE, and a little less than half of the SLE patients had at least 8 factors of social disadvantage.

Although we found significant associations between several individual SDOH and SLE prevalence, we also found a significant association between cumulative SDOH burden and SLE prevalence, which indicates that the burden of SLE can surge with the surge in social disadvantage factors among patients. Often, it can be an interplay and interconnection of several SDOHs, which can affect not only disease presentation but also outcomes^46,47^. Future research on understanding the complex interplay of SDOH on SLE presentation, management, and outcomes, and the mechanisms by which SDOH domains trigger the occurrence of disease is warranted in order to mitigate disparities and, hence, improve outcomes in vulnerable populations.

### Limitations

Several limitations are worth considering. Due to the cross-sectional nature of the study, causality cannot be assessed. Our study presented associations and not causal risk factors of SLE. It is likely that several SDOH including unemployment, Medicaid coverage, or physical activity may be the consequences of SLE and not necessarily the risk factors of SLE. Data in MEPS are self-reported and hence prone to recall bias resulting in under- or over-reporting of the SLE data. However, AHRQ identifies random samples to conduct validation by checking the provider data. Also, validity of self-reported disease in MEPS is relatively high^48^. However, future studies need to assess the validity of identifying chronic conditions which are not commonly occurring from the population-based survey databases. MEPS does not obtain information on SLE subtypes, disease severity or length of the condition. Hence, we could not stratify by disease subtype to examine the prevalence of SDOH by SLE subtype. The prevalence of SLE was very low in our study. Hence, for multivariable analyses, we matched patients with SLE to non-SLE adults on age, sex, and survey year. The methods for scoring and categorization of individual SDOH and SDOH burden used in our study are not validated, however, such methods have been utilized in previously published survey database studies^31,49^. Furthermore, it was not possible to further disaggregate individuals in the ‘other or multiple’ race/ethnicity category due to relatively smaller sample size, particularly among those with SLE. MEPS gathers information on non-institutionalized US adults, and hence, the study findings are not generalizable to the institutionalized population. Future studies should assess an interplay of SDOH and its impact on SLE using a longitudinal study design and with a larger sample of patients with SLE. Despite these limitations, our study is the first step towards broader investigations of both SDOH and biological mechanisms in SLE.

## Conclusion

Our study provided the most recent nationally representative estimates of the prevalence of SLE in the US and found that SLE prevalence has increased from 2017 to 2021. Several SDOH were strongly associated with SLE. The odds of SLE were substantially higher in populations with higher social disadvantage, especially those who are poor, are non-Hispanic Black, who live in Northeast, who have psychological distress, and have Medicaid insurance coverage, in the adjusted analyses. Also, a greater prevalence of SLE exists among individuals with a higher SDOH burden. It is important to identify those with SLE and their ability to manage its symptoms. Patients with SLE and their caregivers likely require significant support and resources to manage this chronic condition. From a provider perspective, these findings suggest that additional care and prevention measures be emphasized in patients with SLE of lower socioeconomic status. Our findings also provide evidence for primary care givers and rheumatologists to evaluate SDOH for patients with SLE and refer them to case managers and/or social workers. From a policy perspective, policies aimed to reduce the burden of poor SDOH in this vulnerable population would be helpful not only to those with SLE, but also more holistically to improve population health and reduce inequalities across the socioeconomic spectrum. Lastly, additional multi-level research should be conducted to more fully identify and address the root causes of the associations between SDOH and SLE to help those in the greatest need to support the management of this disease. Future cohort studies with sufficient follow-up which confirm our study findings on the impact of SDOH in patients with SLE and how it impacts patient outcomes are warranted.

## Data Availability

All data analyzed during the study are publicly available on the website: https://www.meps.ahrq.gov/mepsweb/.

## Appendix 1: Social determinants of health, by domain

**Table:**

	Long description of Items/MEPS Survey Questions	Survey responses	Analytic recode for bivariate and multivariable analyses based on survey responses	Analytic recode for cumulative SDOH burden
Economic stability domain				
Food Insecurity (assessed using 10 questions)*	How often in the last 30 days have you run out of food?	Often true; Sometimes true; Never true	1 = Often true/Sometimes true; 0 = Never true	See the footnote for the food insecurity variable
	How often in the last 30 days the food purchased didn’t last?	Often true; Sometimes true; Never true	1 = Often true/Sometimes true; 0 = Never true	
	How often in the last 30 days could not afford to eat balanced meals?	Often true; Sometimes true; Never true	1 = Often true/Sometimes true; 0 = Never true	
	In the last 30 days did you reduce or skip meals because there wasn’t enough money for food?	Yes; No	1 = Yes; 0 = No	
	How many days in the last 30 days were meals skipped?	0–30 days (continuous response)	1= “>=3 days”; 0=”0–2 days”	
	In the last 30 days did you ever eat less because there wasn’t enough money for food?	Yes; No	1 = Yes; 0 = No	
	In the last 30 days did you ever go hungry because there wasn’t enough money for food?	Yes; No	1 = Yes; 0 = No	
	In the last 30 days did you lose weight because there wasn’t enough money for food?	Yes; No	1 = Yes; 0 = No	
	In the last 30 days did you not eat for a whole day because there wasn’t enough money for food?	Yes; No	1 = Yes; 0 = No	
	How many days in the last 30 days you had not eaten for a whole day because there wasn’t enough money for food?	0–30 days (continuous response)	1= “>=3 days”; 0=”0–2 days”	
Use of food stamps	Did anyone in the household purchase food stamps?	Yes; No	Use (Yes); No use (No)	1 = Use of food stamps; 0 = No use of food stamps;
Employment status	Individuals age 16 and older were asked about employment status	Employed; Job to return; Not employed	Employed (Employed; Job to return); Not employed (Not employed)	1 = Not employed; 0 = Employed
Poverty level	Family income as % of poverty line	Poor/Negative; Near poor; Low income; Middle income; High income	Same as survey responses	0=”Middle income or high income”; 1=”Poor/negative income or near poor income or low income”
Family income level	Family’s total annual income	Continuous response	≤$25,000; $25,001-$50,000; $50,001-$100,000; >$100,000	0=”>$50,000”; 1=”≤$$50,000”
Education domain				
Education level	Number of years of education completed when entering MEPS for individuals 5 years or older	Inapplicable (for individuals < = 5 years of age); No school /kindergarten only; Elementary grades 1–8; high school grades 9–11; grade 12; 1 year college; 2 years college; 3 years college; 4 years college; 5 + years college	≤ High school (No school /kindergarten only, elementary grades 1–8, high school grades 9–11, grade 12); 1–2 years of college (1 year college, 2 years college); 3–4 years of college (3 years college, 4 years college); 5,+ years of college (5 + years college)	0=”At least some college”; 1=”≤ High school”
Social and community context domain				
Race/Ethnicity	Race/ethnicity of the respondents	Hispanic; Non-Hispanic White only; Non-Hispanic Black only; Non-Hispanic Asian only; Non-Hispanic other race or multiple race	Non-Hispanic White (Non-Hispanic White only); Non-Hispanic Black (Non-Hispanic Black only); Hispanic (Hispanic); Other races or multiple races (Non-Hispanic Asian only; Non-Hispanic other race or multiple race)	0=”Non-Hispanic White”; 1=”Other than non-Hispanic Whites”
Marital status	Marital status	Married; Widowed; Divorced; Separated; Never Married	Married (Married); Divorced/Widowed /Separated/Never Married (Widowed; Divorced; Separated; Never Married)	0=”Married”’ 1=”Divorced/Widowed /Separated/Never Married”
Geographic location	Census region	Northeast; Midwest; South; West	Same as survey responses	0=”West”; 1=”Rest of the US geographic regions”
Language spoken	Language spoken at home other than English	Inapplicable (for English); Spanish; Another non-English	English (Inapplicable); Non-English (Spanish; another non-English)	0=”English”; 1=”Non-English”
Time lived in the US/ Nativity	Years lived in the US	Inapplicable (for those born in the US); Less than 1 year; 1-<5 years; 5-<10 years; 10-<15 years; 15,+ years	Born in the US (Response of Inapplicable); <1–10 years (Less than 1 year; 1-<5 years; 5-<10 years); >10 years (10-<15 years; 15,+ years)	0=”Born in the US”; 1=”Rest-Not born in the US”
Neighborhood and built environment domain				
Physical activity	Spends 30 min or more in moderate to vigorous physical activity at least five times a week	Yes; No	Same as survey responses	0=”Yes”; 1=”No”
Smoking status	How often smoke cigarettes? (for age 18 years and above)	Every day; Some days; Not at all	Smoker (Every day; Some days); Non-smoker (Not at all)	0=”Non-smoker”; 1=”Smoker”
Psychological distress**	During the past 30 days, how often you felt nervous?	None of the time; Little of the time; Some of the time; Most of the time; All of the time	0 = None of the time; 1 = Little of the time; 2 = Some of the time; 3 = Most of the time; 4 = All of the time	See the footnote for the psychological distress variable
	During the past 30 days, how often you felt hopeless?	None of the time; Little of the time; Some of the time; Most of the time; All of the time	0 = None of the time; 1 = Little of the time; 2 = Some of the time; 3 = Most of the time; 4 = All of the time	
	During the past 30 days, how often you felt restless or fidgety?	None of the time; Little of the time; Some of the time; Most of the time; All of the time	0 = None of the time; 1 = Little of the time; 2 = Some of the time; 3 = Most of the time; 4 = All of the time	
	During the past 30 days, how often you felt sad that nothing could cheer the person up?	None of the time; Little of the time; Some of the time; Most of the time; All of the time	0 = None of the time; 1 = Little of the time; 2 = Some of the time; 3 = Most of the time; 4 = All of the time	
	During the past 30 days, how often you felt everything was an effort?	None of the time; Little of the time; Some of the time; Most of the time; All of the time	0 = None of the time; 1 = Little of the time; 2 = Some of the time; 3 = Most of the time; 4 = All of the time	
	During the past 30 days, how often you felt worthless?	None of the time; Little of the time; Some of the time; Most of the time; All of the time	0 = None of the time; 1 = Little of the time; 2 = Some of the time; 3 = Most of the time; 4 = All of the time	
Healthcare access and quality domain				
Health insurance type	Type of health insurance coverage during the survey year	< 65 any private; <65 public only; 65 + no Medicare and any public/private; 65 + Medicare only; 65 + Medicare and private; 65 + Medicare and other public only; <65 uninsured, 65 + uninsured	Private < 65 years old (Any private < 65 years of age); Medicare ≥ 65 years old (65 + Medicare only; 65 + Medicare and private; 65 + Medicare and other public only); Medicaid all ages (< 65 public only; 65 + no Medicare and any public/private); Uninsured (< 65 uninsured, 65 + uninsured)	1=”Uninsured”; 0=”Private/Medicare/ Medicaid”
Usual source of care	Does a person have usual source of care provider (doctor’s office, clinic, health center, or other place that an individual usually goes to if they are sick or needs advice about health?	Yes; No	Same as survey responses	1=”No”; 0=”Yes”
Transportation time to usual source of care	How long it takes the person to get to a usual source of care?	Inapplicable (for those who do not have usual source of care); Less than 15 min; 15–30 min; 31–60 min; 61–90 min; 91–120 min; >120 min	≤ 30 min (Inapplicable; Less than 15 min; 15–30 min); 31–90 min (31–60 min; 61–90 min); >90 min (91–120 min; >120 min)	1=”>30 min” 0=”≤30 min”
Unmet medical needs	Whether the person was unable to receive/afford or was delayed in receiving medical care (due to cost)	Yes; No	Same as survey responses	1=”Yes”; 0=”No”
Unmet prescription needs	Whether the person was unable to receive/afford or was delayed in receiving necessary prescription medications (due to cost)	Yes; No	Same as survey responses	1=”Yes”; 0=”No”
Satisfaction with usual source of care provider (assessed using four questions)				
1. Provider asked patient about other treatment	Whether the provider asked the patient about other prescription medications and treatments other doctors may give them	Yes; No	Same as survey responses	1 = No; 0 = Yes
2. Provider asked patient to help decide	Whether the provider asked the patient to help make decisions between a choice of treatments	Yes; No	Same as survey responses	1 = No; 0 = Yes
3. Provider explained options to patient	Whether the provider present and explain all treatment options to the person	Yes; No	Same as survey responses	1 = No; 0 = Yes
4. Provider speaks patient’s language	Whether the provider speaks the person’s language or provide translator services	Yes; No	Same as survey responses	1 = No; 0 = Yes

*Food insecurity variable was created by summing the responses to the 10 survey questions listed above, with value of 1 for each question response indicating food insecurity and 0 indicating no food insecurity. After getting the sum of responses, the variable was categorized as full food security (sum score of 0), marginal food security (sum score of 1–2), low food security (sum score of 3–5), and very low food security (sum score of 6–10). To calculate cumulative SDOH score, for food insecurity variable, a value of 0 was assigned to full or marginal food security, while a value of 1 was assigned to low to very low food security.

**Psychological distress variable was created by summing the response scores for a total of possible score range of 0 to 24. Based on the score, psychological distress was categorized as no psychological distress (score of 0), without serious psychological distress (score of 1–12), and serious psychological distress (score of 13–24). To calculate cumulative SDOH score, for psychological distress variable, a value of 0 was assigned to no psychological distress, while a value of 1 was assigned to with or without serious psychological distress.

## Abbreviations

SLE: Systemic lupus erythematosus
SDOH: Social determinants of health
MEPS: Medical expenditure panel survey
AOR: Adjusted odds ratio
US: United States
AHRQ: Agency for healthcare research and quality
NCHS: National center for health statistics
ICD-10-CM: International classification of diseases, tenth edition, clinical modification
EULAR: European alliance of associations for rheumatology
CI: Confidence intervals
